# Evaluation of population impact of candidate polymorphisms for coronary heart disease in the Framingham Heart Study Offspring Cohort

**DOI:** 10.1186/1753-6561-3-s7-s118

**Published:** 2009-12-15

**Authors:** Yu Yan, Yijuan Hu, Kari E North, Nora Franceschini, Danyu Lin

**Affiliations:** 1 Department of Epidemiology, University of North Carolina, 2101 McGavran-Greenberg Hall, Chapel Hill, North Carolina 27599, USA; 2 Department of Biostatistics, University of North Carolina, 3101 McGavran-Greenberg Hall, Chapel Hill, North Carolina 27599, USA; 3 Carolina Center for Genome Sciences, University of North Carolina, 5009 Genetic Medicine Building, Chapel Hill, North Carolina 27599, USA

## Abstract

In order to evaluate the population impact of putative causal genetic variants over the life course of disease, we extended the static estimation of population-attributable risk fraction and developed a novel tool to evaluate how the population impact changes over time using the Framingham Heart Study Offspring Cohort data provided to the Genetic Analysis Workshop 16, Problem 2. A set of population-attributable risk fractions based on survival functions were estimated under the proportional hazards models. The development of this novel measure of population impact creates a more comprehensive estimate of population impact over the life course of disease, which may help us to better understand genetic susceptibility at the population level.

## Background

The ongoing discovery of new genetic markers from genome-wide association studies presents opportunities and challenges for scientists to evaluate these new biomarkers. One of the critical questions that has been raised is how to evaluate the potential population impact of these new markers. First proposed by Levin in 1953 [1], the primary measure of impact is the population-attributable risk fraction (PAF, also known as the population-attributable risk proportion). The PAF, determined by the prevalence of exposure and the magnitude of association, measures the proportion of disease risk in the total population associated with one or multiple exposures; thus the PAF is useful in evaluating the impact of different exposures at the population level. However, the current PAF estimation does not account for age of onset data (i.e., time-to-event data). In this study, we developed methodological approaches to estimate the population impact of genetic variants over the life course of disease using the longitudinal Framingham Heart Study Offspring Cohort and incident coronary heart disease (CHD) events.

## Methods

### Population and phenotype

We used the Framingham Heart Study Offspring Cohort data provided to the Genetic Analysis Workshop (GAW) 16, Problem 2, for the analyses. The Framingham Heart Study is a longitudinal community-based cohort of cardiovascular disease and its risk factors that began in 1948 with the recruitment of the Original Cohort [2]. Between 1971 and 1975, 5124 children or spouses of the Original Cohort were enrolled into the Offspring Cohort [3]. The Offspring Cohort has undergone eight examinations every 4 to 8 years. The present study is composed of unrelated Offspring participants. Of 2760 Offspring participants who gave informed consent for data collected to be used by anyone, we excluded those biologically related participants (*n *= 813), participants without genotyping data (*n *= 211), and those with prevalent CHD at baseline (*n *= 2). After these exclusions, a total of 1734 unrelated Offspring participants were available for analysis. The Framingham Heart Study Offspring Cohort study protocol was approved by Boston University Medical Center Institutional Review Board and this investigation was approved by University of North Carolina at Chapel Hill Institutional Review Board.

A CHD event was defined as any of the following: recognized myocardial infarction diagnosed through an electrocardiogram or enzymes, coronary insufficiency, or death attributed to CHD.

### Genotyping methods and single-nucleotide polymorphism (SNP) selection

The Affymetric 500 k chip was used to genotype individual participant DNA. SNPs selected for this study were based on published candidate gene studies and genome-wide association studies. A total of 23 SNPs associated with major CHD or major cardiovascular disease were included in this investigation.

### Statistical analyses

To assess whether genotype distributions departed from Hardy-Weinberg equilibrium, a *χ*^2 ^goodness-of-fit test was used. We used Cox proportional hazards to estimate the hazard ratios and 95% confidence intervals of incident CHD. The hazard function was formulated on the age scale using the age at onset of CHD obtained as part of the GAW 16 Problem 2 data release. Covariates, including sex, smoking, diabetes, systolic blood pressure, anti-hypertensive treatment, total cholesterol levels, high-density lipoprotein cholesterol, and body mass index, were included in the models to reduce the residual variance. The association was considered to be significant if the *p*-value was less than 0.05. Assuming additive inheritance, a variable taking on the values 0 for reference genotype, 1 for heterozygous genotype, and 2 for homozygous genotype was used to test genetic effects for each SNP.

Significant associations between three SNPs (rs1333049, rs618675, and rs1376251) and increased risk of incident CHD were noted. We further explored the association with the risk score, which was constructed by summing the number of risk alleles across these three CHD susceptibility SNPs. The distribution ranged from zero to six alleles. Because very few participants have zero (*n *= 27) or six (*n *= 8) risk alleles, these participants were included into the closest group (e.g., zero was grouped with one risk allele).

Our methodological approach integrates multiple PAF estimates at multiple ages for a single variant in an attempt to create a comprehensive estimate of population impact.

For a binary disease status *D *and a binary exposure indicator *E*, the PAF is defined as

[1]. We extended this static measure to the age-of-onset data with potentially multiple risk factors

where *T *denotes the time to disease and *X *denotes a set of potential genetic factors. We can rewrite this measure in terms of survival function

and *S*(*t*) = Pr(*T *> *t*) and *S*_0_(*t*) = Pr(*T *> *t *| *X *= 0).

*S*(*t*) was estimated by the Kaplan-Meier nonparametric method. If *X *pertained to a single genetic variant, then we estimated *S*_0_(*t*) by the Kaplan-Meier method as well. If *X *consisted of several genetic factors, then we estimated *S*_0_(*t*) under a semiparametric regression model. All the statistical analyses were performed in SAS 9.1 (SAS institute, Cary, NC). A PAF plot was provided to indicate how the population impact changed over the life course of disease.

## Results

A total of 137 incident CHD events were identified. The allele frequencies for all 23 SNPs analyzed in this study were in Hardy-Weinberg equilibrium (*p *> 0.01). Because the estimates for SNP effects on incident CHD were almost identical after adjusting for aforementioned covariates, only unadjusted hazard ratios with 95% confidence intervals were reported (Table 1). Three SNPs (rs1333049 close to the *CDKN2A/2B *gene, rs618675 in the *GJA4 *gene, and rs1376251 in the *TAS2R50 *gene) were significantly associated with the incident CHD (Table 1). Further exploration indicated that the risk score (*p *= 0.0004) was significantly associated with incident CHD risk (Table 1).

**Table 1:** Characteristics of selected SNPs, and associations between the CHD incidence and SNPs

							HR estimates (95% CI)		
							
Closest gene	Gene name	SNP [reference]	Chr	Physical position	Risk allele	MAF	Heterozygous genotype	Homozygous genotype	*p*-Value
*GJA4*	gap junction protein, alpha 4	rs618675 [4]	1	34922761	C	0.19	1.32 (1.01, 1.72)	1.73 (1.01, 2.97)	0.0451
*PCSK9*	proprotein convertase subtilisin/kexin type 9	rs2114580 [4]	1	55167236	A	0.24	1.22 (0.94, 1.59)	1.49 (0.88, 2.54)	0.1376
*PSRC1*	proline/serine-rich coiled-coil 1	rs599839 [5]	1	109623689	A	0.22	1.05 (0.79, 1.41)	1.11 (0.62, 1.98)	0.7262
*MIA3*	melanoma inhibitory activity family, member 3	rs17465637 [5]	1	220890152	C	0.29	1.07 (0.82, 1.39)	1.14 (0.67, 1.93)	0.6282
*FMN2*	formin 2	rs17672135 [6]	1	238512219	C	0.11	1.20 (0.83, 1.73)	1.44 (0.69, 2.99)	0.3286
*OR13G1*	olfactory receptor, family 13, subfamily G, member 1	rs1151640 [7]	1	245902573	C	0.43	0.98 (0.77, 1.25)	0.96 (0.59, 1.56)	0.8628
		rs2943634 [5]	2	226776324	C	0.35	1.08 (0.84, 1.39)	1.16 (0.71, 1.92)	0.5508
		rs10516882 [4]	4	92127599	C	0.17	1.19 (0.85, 1.67)	1.41 (0.72, 2.78)	0.3170
*THBS4*	thrombospondin 4	rs264986 [4]	5	79206180	T	0.27	1.01 (0.77, 1.32)	1.01 (0.59, 1.74)	0.9656
*MTHFD1L*	methylenetetrahydrofolate dehydrogenase 1-like	rs6922269 [5]	6	151294678	A	0.27	1.13 (0.87, 1.48)	1.29 (0.76, 2.18)	0.3506
*PHACTR1*	phosphatase and actin regulator 1	rs1512411 [4]	6	13439076	C	0.34	1.01 (0.78, 1.31)	1.03 (0.61, 1.71)	0.9235
*WNT2*	wingless-type MMTV integration site family member 2	rs39312 [4]	7	116742021	A	0.35	1.06 (0.83, 1.35)	1.12 (0.68, 1.83)	0.6554
*CDKN2A/2B*	cyclin-dependent kinase inhibitor 2A/2B	rs1333049 [6]	9	22115503	C	0.48	1.28 (1.01, 1.63)	1.64 (1.02, 2.66)	0.0433
		rs501120 [5]	10	44073873	C	0.14	0.99 (0.71, 1.39)	0.99 (0.50, 1.94)	0.9681
*KIAA0528*	KIAA0528	rs10505879 [4]	12	22539123	G	0.22	1.13 (0.86, 1.50)	1.28 (0.74, 2.24)	0.3753
*TAS2R50*	taste receptor, type 2, member 50	rs1376251 [7]	12	11030119	C	0.33	1.31 (1.00, 1.71)	1.71 (1.01, 2.92)	0.0476
*ALOX5AP*	arachidonate 5-lipoxygenase-activating protein	rs7984952 [4]	13	30129806	T	0.43	1.24 (0.97, 1.58)	1.54 (0.95, 2.51)	0.0817
		rs7995384 [4]	13	30177259	T	0.29	1.20 (0.92, 1.57)	1.44 (0.84, 2.48)	0.1830
		rs117395 [4]	13	30365911	T	0.13	1.09 (0.75, 1.59)	1.19 (0.56, 2.52)	0.6459
*SMAD3*	SMAD family member 3	rs17228212 [5]	15	65245693	C	0.29	1.04 (0.80, 1.35)	1.08 (0.64, 1.81)	0.7781
		rs2549513 [4]	16	78108228	C	0.13	1.03 (0.73, 1.47)	1.06 (0.53, 2.15)	0.8617
*CDH13*	cadherin 13, H-cadherin	rs8055236 [6]	16	81769899	T	0.19	1.26 (0.95, 1.67)	1.59 (0.91, 2.78)	0.1045
*SEZ6L*	seizure related 6 homolog	rs688034 [6]	22	25019635	C	0.33	1.10 (0.84, 1.43)	1.20 (0.71, 2.04)	0.4971
Risk Score^a^							1.32 (1.13, 1.54)		0.0004

^a^The HR estimated the hazard of having incident CHD for one unit increase in the risk score

**Figure 1:**
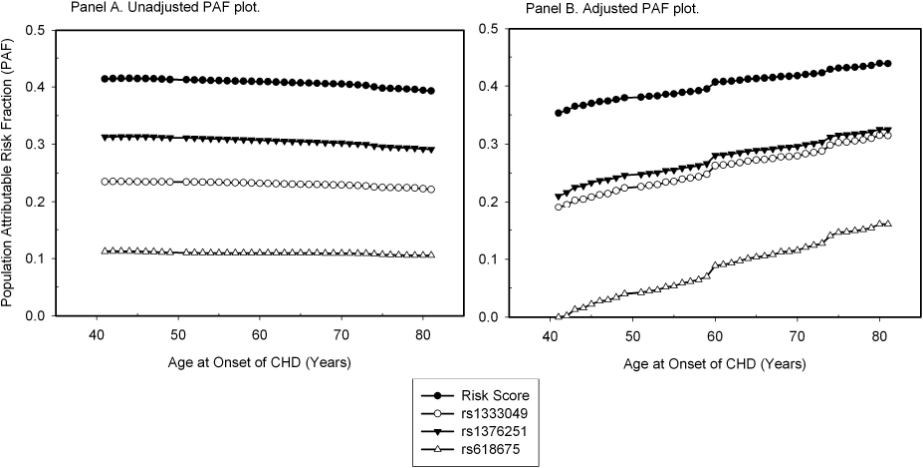
**The PAF plot over time for the risk score, rs1333049, rs1376251, and rs618675**.

The PAF plots for the risk score and three significant SNPs with and without adjustment for covariates are shown in Figure 1. The age at onset of CHD ranged from 41 to 81 years old. The PAFs were much higher for the risk score (PAF = 41% on average) than for each individual SNP. The unadjusted PAFs showed a subtle decline with age, whereas the adjusted PAFs slightly increased over time.

## Discussion

Our study replicates the association between CHD risk and rs1333049 close to the *CDKN2A/2B *gene, rs618675 in the *GJA4 *gene, and rs1376251 in the *TAS2R50 *gene, in Caucasians. However, the number of events (maximum of 137 for CHD) was small. Thus, we had limited power to detect association for each individual SNP and our study results need to be validated in different, large population-based studies.

We assessed here the impact of the known cardiovascular disease genes/loci on the population burden of CHD over time, based on data from the Framingham Heart Study Offspring Cohort. Static PAFs have been extensively used to rank risk factors and to assess the prospective gains in disease prevention. In this study, we extended the static estimation of PAFs and evaluated how the population impact of genetic variants changed over the life course of CHD, as shown in the PAF plot (Figure 1). The unadjusted PAFs associated with genetic variants slightly decreased as age advanced, whereas adjusted PAFs showed a subtle increase with age, which may be due to the small number of events in these data, especially in the early and late age groups. For example, only six CHD events occurred before the age of 45, whereas eight events occurred after 75. However, we observed much higher PAFs for the risk score compared with each individual SNP, suggesting the importance of evaluating multiple genetic variants for the population impact analysis.

While the use of the risk score summary metric was useful in this population in which no single SNP achieved a large PAF, these estimates should be interpreted with caution because any time we combine SNP effects based on statistical significance and effect size, we will automatically obtain an improved effect estimate and *p*-value.

## Conclusion

Our development of the novel tool for population impact extends the current PAF analyses and creates a more comprehensive estimate of population impact over the life course of disease, which may improve the understanding of genetic risk factors at the population level.

## List of abbreviations used

CHD: Coronary heart disease; GAW: Genetic Analysis Workshop; HR: Hazard ratio; MAF: Minor allele frequency; PAF: Population-attributable risk fraction; SNP: Single-nucleotide polymorphism.

## Competing interests

The authors declare that they have no competing interests.

## Authors' contributions

YY and KEN conceived of the study, performed the statistical analysis, and drafted the manuscript. YH helped performed the statistical analysis and helped to draft the manuscript. NF participated in the study design and helped to draft the manuscript. DL participated in the study design, helped performed the statistical analysis and helped to draft the manuscript. All authors read and approved the final manuscript.
